# The prevalence of sarcopenia in spondyloarthritis patients: a meta-analysis

**DOI:** 10.1186/s13075-024-03299-5

**Published:** 2024-03-16

**Authors:** Jiawen Hu, Yiwen Wang, Xiaojian Ji, Yinan Zhang, Kunpeng Li, Feng Huang

**Affiliations:** 1 Department of Rheumatology and Immunology, The First Medical Center, Chinese PLA General Hospital, 28 Fuxing Road, Haidian District, Beijing, 100853 China; 2 Medical School of Chinese PLA, 28 Fuxing Road, Haidian District, Beijing, 100853 China

**Keywords:** Sarcopenia, Spondyloarthritis, Prevalence, Meta-analysis

## Abstract

**Background:**

Spondyloarthritis (SpA) is a chronic inflammatory disorder that affects sacroiliac joints and spine, resulting in substantial disability. Sarcopenia, characterized by the loss of muscle mass and function, is a prevalent comorbidity in various chronic diseases. However, the exact prevalence of sarcopenia in SpA patients remains uncertain. The objective of this study is to conduct a systematic review and meta-analysis of the available literature to determine the prevalence of sarcopenia in SpA.

**Methods:**

A comprehensive search was conducted in EMBASE, MEDLINE, WEB OF SCIENCE, and COCHRANE databases to identify relevant studies published up to 2023. Studies investigating the prevalence of sarcopenia in SpA patients were included. Data on study characteristics, participant demographics, diagnostic criteria for sarcopenia, and prevalence rates were extracted. Meta-analysis was performed using a random-effects model to estimate the overall prevalence of sarcopenia in SpA patients.

**Results:**

A total of 16 studies that met the inclusion criteria were included in the systematic review. These studies encompassed a combined sample size of 999 patients with SpA. The meta-analysis findings revealed that the overall prevalence of sarcopenia in SpA patients was 25.0% (95% confidence interval: 0.127 to 0.352). Furthermore, the prevalence of presarcopenia and severe sarcopenia was found to be 21.0% and 8.7%, respectively. Subgroup analysis was conducted to examine different diagnostic criteria, subtypes, and sex of SpA in relation to sarcopenia.

**Conclusion:**

This systematic review and meta-analysis provide a comprehensive overview of the prevalence of sarcopenia in SpA patients. The findings suggest a high prevalence of sarcopenia in SpA patients, emphasizing the need for targeted interventions to prevent and manage sarcopenia. And further research is needed to explore the underlying mechanisms and potential therapeutic strategies for sarcopenia in SpA.

**Supplementary Information:**

The online version contains supplementary material available at 10.1186/s13075-024-03299-5.

## Introduction

Spondyloarthritis (SpA) is a chronic inflammatory disease that primarily affects the axial skeleton, including the spine and sacroiliac joints [1]. It is characterized by chronic pain, stiffness, and progressive joint damage, leading to functional disability and reduced quality of life. SpA encompasses a range of diseases, including ankylosing spondylitis (AS), psoriatic arthritis (PsA), reactive arthritis, arthritis of inflammatory bowel disease, a subgroup of juvenile idiopathic arthritis (JIA), and undifferentiated spondyloarthritis. Slimily, SpA patients often experience a range of extra-articular manifestations, such as uveitis, psoriasis, and inflammatory bowel disease. In addition to the musculoskeletal symptoms, growing evidence suggests that SpA patients are also at risk of developing sarcopenia [2]. Sarcopenia is a condition characterized by the loss of skeletal muscle mass, strength, and physical function [3]. While it is commonly associated with aging, emerging research has demonstrated that chronic inflammation could contribute to the development of sarcopenia [4].

The chronic inflammatory state in SpA is driven by immune cell dysregulation and the release of pro-inflammatory cytokines, including C-reactive protein, tumor necrosis factor (TNF)-alpha, and interleukin-6 [5]. These inflammatory mediators have been found to contribute to muscle wasting and hinder muscle regeneration, ultimately leading to the development of sarcopenia [6]. These studies have demonstrated the potential occurrence of sarcopenia in SpA patients. Moreover, sarcopenia is associated with decreased physical function, increased disability, and higher healthcare utilization. Additionally, the presence of sarcopenia in SpA patients can negatively impact treatment response and increase the risk of comorbidities, such as falls and fractures.

Despite the potential clinical significance of sarcopenia in SpA, there is currently limited evidence regarding its prevalence in this specific patient population. Previous studies have reported varying prevalence rates, underscoring the need for a systematic review and meta-analysis to consolidate the available data and gain a more comprehensive understanding of the burden of sarcopenia in different subtypes of SpA. Therefore, the objective of this study is to determine the prevalence of sarcopenia in SpA patients. By synthesizing the existing literature, this study aims to provide valuable insights into the impact of sarcopenia on SpA patients and contribute to the development of future research and clinical management strategies.

## Methods

This systematic review and meta-analysis was implemented according to the Preferred Reporting Items for Systematic Review and Meta-Analysis (PRISMA) guidelines. And the protocol was registered on the International Prospective Register of Systematic Reviews (PROSPERO) as CRD42023464459.

### Data sources and search strategy

A comprehensive search was performed to search the relevant studies published from inception dates to September 26, 2023 in electronic databases including Embase, Medline, Web of science and Cochrane. The search strategy was conducted using a combination of relevant keywords and medical subject headings terms, such as [‘sarcopenia’ OR ‘muscle mass’ OR ‘hand strength’ OR ‘walking speed] and [‘spondylarthropathies’ OR ‘spondylitis, ankylosing’ OR ‘arthritis, psoriatic’ OR ‘arthritis, infectious’ OR ‘arthritis, reactive’ OR ‘arthritis, juvenile’ OR ‘enthesitis related arthritis’ OR ‘arthritis AND inflammatory bowel disease’]. The reference lists of included studies and relevant review articles were also manually searched to identify any possible appropriate studies.

### Study selection

Two independent investigators screened the titles and abstracts of all identified articles to exclude articles irrelevant to the systematic review. The second step was to independently read the full-text articles of potentially eligible studies and then retrieved and assessed the studies that met the inclusion criteria. Any discrepancies were resolved through discussion and consensus. The inclusion and exclusion criteria were as follows. Cross section survey, case-control study and cohort study were included if they recorded the prevalence of sarcopenia, presarcopenia or severe sarcopenia in SpA patients or provided sufficient data to calculate it. Meanwhile, these studies should have used validated diagnostic criteria for sarcopenia, presarcopenia or severe sarcopenia and for SpA (the 2009 ASAS criteria [7]). In cases where multiple articles had identical participants, only the latest study was selected for review. Editorials, letters, reviews, case reports and case series were excluded. Studies conducted on animal models and not published in English were also excluded.

### Data extraction

Data were extracted from the included studies using a standardized data extraction form, including study characteristics (author, year, country, study design), patient characteristics (sample size, age, sex, body mass index (BMI), disease duration, assessment method for muscle mass, muscle mass, grip strength, physical performance), diagnostic criteria and prevalence of sarcopenia, presarcopenia or severe sarcopenia. Two independent investigators also extracted the subtype of SpA, questionnaires and the cut-off points of muscle mass, grip strength and gait speed. In cohort study, the two independent investigators particularly extracted the time of follow up, interventions and outcomes.

### Quality assessment

The quality of included studies was assessed using the risk of bias tool for prevalence studies by Newcastle-Ottawa Scale (NOS) [8]. The NOS includes 10 questions and evaluates both external (Questions 1 to 4) and internal (Questions 5 to 10) validity. Besides, each study was assigned an overall risk of study bias scored as low, moderate or high risk (Question 11). Two independent investigators gave their judgment to each question with the yes indicating low risk and no indicating high risk. Studies with greater than or equal to 8 questions scored as low risk would be low risk, those with 6 to 7 questions scored as low risk would be moderate risk, and those with less than or equal to 5 questions scored as low risk would be high risk. Any discrepancies were resolved through discussion and consensus.

### Statistical analysis

All analyses were performed by Stata 17 statistical software. The metaprop command in Stata was used to calculate the overall pooled estimates prevalence and its 95% confidence interval (CI) with inverse-variance weights obtained from random-effect meta-analysis models. The prevalence of sarcopenia in SpA was calculated by dividing the number of sarcopenia patients by the total number of SpA patients in each study. When one study reported more than one classification to calculate prevalence, only the more widespread accepted was kept. For subgroup analysis, classifications, subtypes and sex were conducted to explore potential sources of heterogeneity, and I2 statistic was used to assess it. To be specific, the prevalence was estimated by classifications (European Working Group on Sarcopenia in Older People (EWGSOP) [9], EWGSOP2 [10], Asian Working Group for Sarcopenia (AWGS) [11], Baumgartner [12], Lee’s Equation [13] and others), subtypes of SpA (AS, PsA and SpA) and sex (men and women).

## Results

### Search results

**Fig. 1:**
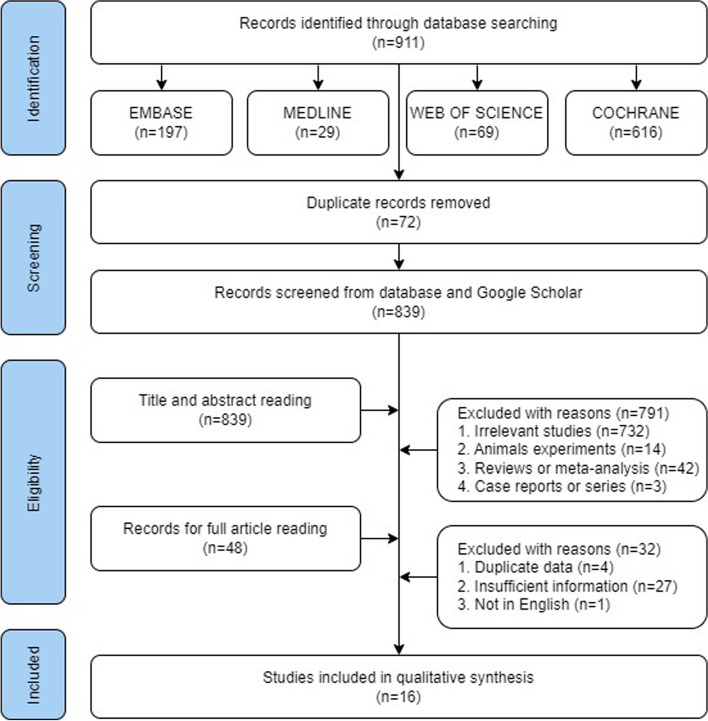
Flow diagram for systematic review and meta-analysis

A comprehensive search was conducted, and the initial search identified 911 records (197 in Embase, 29 in Medline, 69 in Web of science and 616 in Cochrane). After removing duplicates (*n* = 72), 839 titles and abstracts were screened. Of these, a total of 48 relevant studies were selected for a full-text review for the eligibility assessment. Following the application of the inclusion and exclusion criteria, 16 studies were finally included in this systematic review (Fig. 1) [2, 14–28].

### Description of studies

A total of 16 studies and 999 SpA patients were included in this systematic review (Table 1). Overall, studies were conducted between 2013 and 2023, and 9 studies were carried out in Europe, 3 in South American, 3 in Asia and 1 in Africa. Dual energy X-ray absorptiometry (DXA) was the most often used method to assess muscle mass (10 studies), followed by bioelectric impedance analysis (BIA) (4 studies), all of them were corrected by height squared. Most of the studies were cross section surveys (9 studies), 4 were case-control studies, 2 were cohort studies and only 1 were diagnosis test (Table 2). In one cohort studies, anti-TNF was used and sarcopenia reversion after 24 months. In the other study, the researcher chose Ustekinumab for patients and saw the decreased in total lean mass after 6 months. There were subtle differences in the cut off points for muscle mass, grip strength and gait speed according to its classification. In terms of questionnaires, 4 of these studies used health assessment questionnaire (HAQ), 2 used Ankylosing Spondylitis quality of life questionnaire (ASQoL), 1 used international physical activity questionnaire (IPAQ) and general practice physical activity questionnaire (GPPAQ), respectively. Only 1 used the sarcopenia and quality of life (SarQoL), which was particularly designed for sarcopenia [29].

**Table 1:** Characteristics of the studies included in the systematic review

Study	Country/Region	Type of study	Classification used	Assessment method for muscle mass	Correction method for muscle mass	Age (mean (SD)) (total-men-women)	BMI (mean (SD)) (total-men-women)	Disease duration (m, mean (SD)) (total-men-women)	Sample (n) (total-men-women)	Muscle mass (mean (SD)) (total-men-women)	Grip strength (mean (SD)) (total-men-women)	Physical performance (mean (SD)) (total-men-women)
Aguiar 2014 [14]	Portugal/Europe	Case-control study	Lee’s equation	Measuring Tape and Skinfold Caliper	Sex, Age, and Ethnicity	45.5 (13.4)-NA-NA	NA	10.9 (11.6)-NA-NA	60-29-31 AS 36-NA-NA PsA 24-NA-NA	7.7 (1.0)-7.8 (0.9) -7.5 (1.0)	NA	NA
Barone 2018 [15]	Italy/Europe	Cross section survey	EWGSOP for sarcopenia Other for presarcopenia	BIA 101	Height	AS 51.6 (8.8)-NA-NA PsA 55.3 (9.1)-NA-NA	AS 24.9 (2.5)-NA-NA PsA 25.6 (3.0)-NA-NA	AS 14.5 (8.4)-NA-NA PsA 11.1 (8.1)-NA-NA	AS 22-14-8 PsA 70-32-38	NA	NA	NA
Barros 2012 [16]	Brazil/South American	Cohort study	Baumgartner	DXA	Height	NA	25.7 (NA)-NA-NA	NA	30-24-6	NA	NA	NA
Fitzgerald 2017 [17]	Ireland/Europe	Cross section survey	Other	BIA	Height	50.8 (11.1)-51 (10.5) -50.4 (13.7)	28.8 (6.3)-29 (6.9) -27.8 (3.4)	24 (11.7)-24.5 (11.6) -22.2 (12.7)	43-34-9	8.7 (1.7)-9.2 (1.6) -7.0 (0.6)	NA	NA
Kavadichanda 2022 [18]	India/Asia	Cross section survey	AWGS	DXA	Height	SpA 38.8 (7.5)-NA-NA PsA 41.3 (9.3)-NA-NA	SpA 23.6 (NA)-NA -NA PsA 25.1 (NA)-NA-NA	NA	SpA 58-44-14 PsA 56-28-28	Total 6.9 (1.1)-NA -NA	NA	NA
Krajewska 2017 [19]	Poland/Europe	Case-control study	EWGSOP	BIA (InBody170)	Height	65.6 (5.9)-NA-NA	30.1 (5.8)-NA-NA	11.1 (8.9)-NA-NA	51-0-51	6.44 (0.72)	NA	NA
Leite 2020 [20]	Brazil/South American	Cross section survey	Other	DXA	Height	NA	NA	NA	96-43-53	7.7 (1.3)-8.54 (1.29) -6.94 (0.87)	NA	NA
Maghraoui 2016 [21]	Morocco/Africa	Cross section survey	EWGSOP for sarcopenia Baumgartner for presarcopenia	DXA	Height	40.9 (11.0)-NA-NA	25.3 (4.0)-NA-NA	9.3 (7.9)-NA-NA	67-67-0	7.4 (0.8) -NA-NA	NA	NA
Merle 2023 [2]	France/Europe	Case-control study	EWGSOP2	DXA	Height	47.1 (13.7)-47.0 (13.6) -47.3 (13.9)	26.0 (5.0)-25.8 (4.8) -26.2 (5.2)	12.2 (9.0)-11.9 (10.4) -12.6 (7.6)	103-50-53	7.4 (1.3)-8.0 (1.2) -6.6 (1.0)	28.8 (13.1)-37.1 (12.9)-20.8 (6.9)	0.9 (0.4)-0.9 (0.4)-0.9 (0.4)
Neto 2022 [22]	Portugal/Europe	Cross section survey	EWGSOP2	BIA (InBody770)	Height	37.0 (7.0)-NA-NA	25.0 (NA)-NA-NA	7 (3.0)-NA-NA	27-18-9	NA	NA	0.8 (0.1)-NA-NA
Paccou 2020 [23]	France/Europe	Cohort study	Baumgartner	DXA	Height	NA	NA	NA	30-NA-NA	NA	NA	NA
Sequeira 2013 [24]	Portugal/Europe	Case-control study	Lee’s equation	Measuring Tape and Skinfold Caliper	Sex, Age, and Ethnicity	45.5 (13.4)-NA-NA	NA	10.9 (11.6)-NA-NA	60-29-31	NA	NA	NA
Song 2017 [25]	Sourth Korea/Asia	Cross section survey	AWGS Korea criteria	DXA	Height	NA	NA	NA	60-60-0	NA	NA	NA
Soto 2023 [26]	Brazil/South merican	Diagnosis test	ASMM	DXA	Height	49.2 (14.2)-NA-NA	NA	NA	42-24-18 AS 35-NA-NA PsA 7-NA-NA	NA	NA	NA
Tournadre 2017 [27]	France/Europe	Cross section survey	EWGSOP	DXA	Height	SpA 44.1 (12.0) PsA 54.6 (11.0)	SpA 28.7 (5.1) PsA 54.6 (11.0)	SpA 6.4 (9.4) PsA 5.5 (6.8)	SpA 63-NA-NA PsA 11-NA-NA	SpA 8.1 (1.6) PsA 8.0 (1.7)	NA	NA
Younis 2021 [28]	Iraq/Asia	Cross section survey	EWGSOP	DXA	Height	36.6 (7.7)-NA-NA	28.4 (6.3)-NA-NA	6.9 (5.0)-NA-NA	50-47-3	8.0 (1.0)-NA-NA	32.9 (8.3)-NA-NA	0.9 (0.2)-NA-NA

**Table 2:** SpA subtypes, cut-off points, questionnaires and outcomes included in the systematic review and meta-analyses

Study	SpA subtypes	Classification used	Assessment method for muscle mass	Correction method for muscle mass	Cut-off points muscle mass		Cut-off points grip strength		Cut-off points gait speed		Questionnaires	Time of follow up	Interventions	Outcomes
					Men	Women	Men	Women	Men	Women				
Aguiar 2014 [14]	AS, PsA	Lee’s equation	Measuring Tape and Skinfold Caliper	Sex, Age, and Ethnicity	< 10.75 kg/m^2^	< 6.75 kg/m^2^	NA	NA	NA	NA	HAQ	NA	NA	Ultrasound can diagnosis sarcopenia in spondyloarthritis
Barone 2018 [15]	AS, PsA	EWGSOP for sarcopenia Other for presarcopenia	BIA 101	Height	< 10.75 kg/m^2^	< 6.75 kg/m^2^	< 30 kg	< 20 kg	NA	NA	NA	NA	NA	NA
Barros 2012 [16]	active AS	Baumgartner	DXA	Height	< 7.26 kg/m^2^	< 5.45 kg/m^2^	NA	NA	NA	NA	ASQoL	6 m, 12 m, 24 m	Anti-TNF	Sarcopenia reversion (BL: 16.6% vs. 6 m: 13.3% vs. 12 m: 6.6% vs. 24 m: 0%, *p* < 0.001)
Fitzgerald 2017 [17]	axSpA	Other	BIA	Height	< 8.87 kg/m^2^	< 6.42 kg/m^2^	NA	NA	NA	NA	ASQoL	NA	NA	NA
Kavadichanda 2022 [18]	SpA, PsA	AWGS	DXA	Height	< 7.0 kg/m^2^	< 5.4 kg/m^2^	NA	NA	NA	NA	NA	NA	NA	NA
Krajewska 2017 [19]	PsA	EWGSOP	BIA (InBody170)	Height	NA	< 5.45 kg/m^2^	NA	NA	NA	NA	NA	NA	NA	NA
Leite 2020 [20]	PsA	Other	DXA	Height	NA	NA	NA	NA	NA	NA	HAQ	NA	NA	NA
Maghraoui 2016 [21]	AS	EWGSOP for sarcopenia Baumgartner for presarcopenia	DXA	Height	< 7.25 kg/m^2^	NA	< 30 kg	NA	Timed get-up-and-go test > 10s	NA	NA	NA	NA	NA
Merle 2023 [2]	SpA	EWGSOP2	DXA	Height	< 7.0 kg/m^2^	< 5.5 kg/m^2^	< 27 kg	< 16 kg	≤ 0.8 m/s	≤ 0.8 m/s	SarQoL®	NA	NA	NA
Neto 2022 [22]	axSpA	EWGSOP2	BIA (InBody770)	Height	< 7.0 kg/m2	< 6 kg/m^2^	< 27 kg	< 16 kg	≤ 0.8 m/s	≤ 0.8 m/s	IPAQ	NA	NA	NA
Paccou 2020 [23]	active PsA	Baumgartner	DXA	Height	< 7.26 kg/m^2^	< 5.5 kg/m^2^	NA	NA	NA	NA	NA	6 m	Ustekinumab	Total lean mass decreased
Sequeira 2013 [24]	SpA	Lee’s equation	Measuring Tape and Skinfold Caliper	Sex, Age, and Ethnicity	NA	NA	NA	NA	NA	NA	HAQ	NA	NA	NA
Song 2017 [25]	AS	AWGS Korea criteria	DXA	Height	< 7.0 kg/m^2^ NA	< 5.4 kg/m^2^ NA	< 28 kg NA	< 18 kg NA	< 1.0 m/s NA	< 1.0 m/s NA	NA	NA	NA	NA
Soto 2023 [26]	SpA	ASMM	DXA	NA	NA	NA	NA	NA	NA	NA	NA	NA	NA	NA
Tournadre 2017 [27]	SpA, PsA	EWGSOP	DXA	Height	< 7.26 kg/m^2^	< 5.45 kg/m^2^	NA	NA	NA	NA	HAQ	NA	NA	NA
Younis 2021 [28]	AS	EWGSOP	DXA	Height	< 7.0 kg/m^2^	< 6 kg/m^2^	< 27 kg	< 16 kg	≤ 0.8 m/s	≤ 0.8 m/s	GPPAQ	NA	NA	NA

### Study quality

**Fig. 2:**
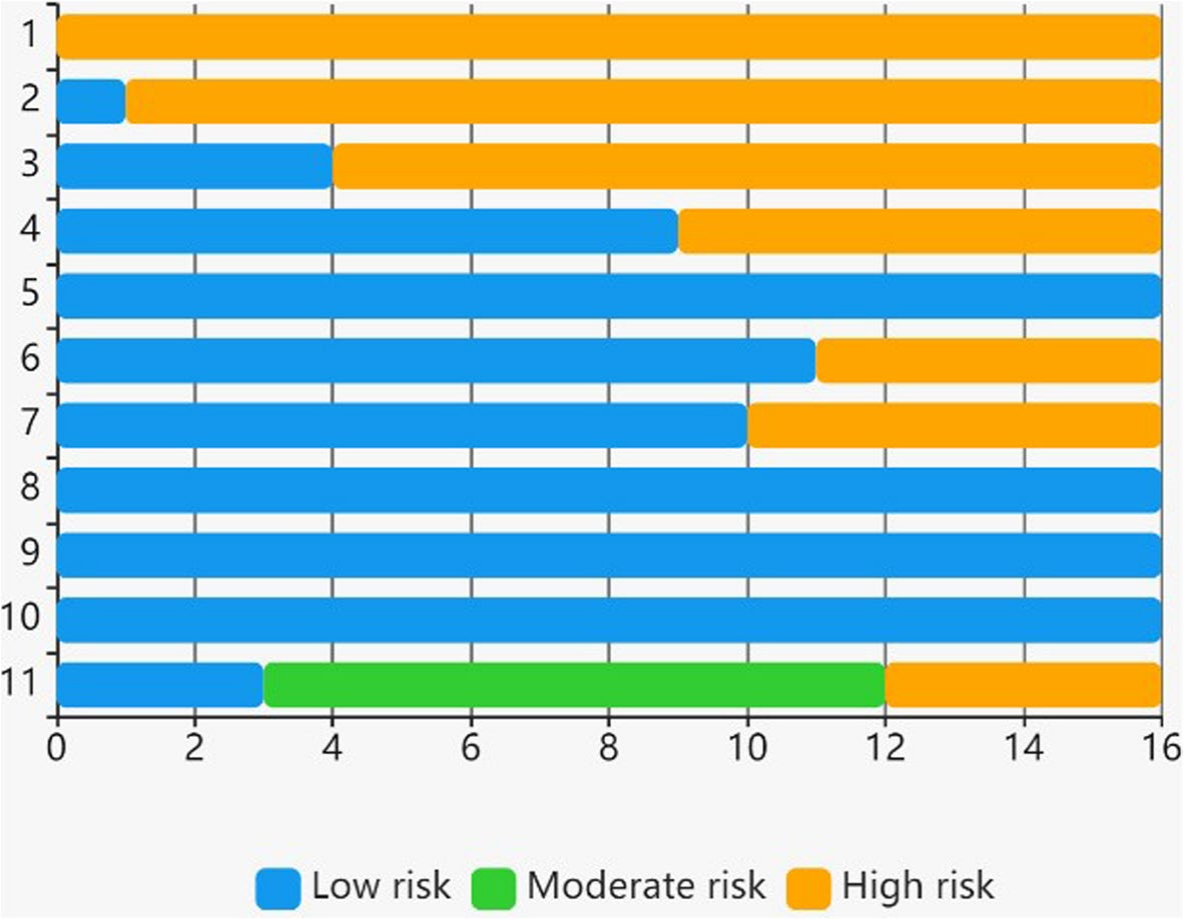
Distribution proportions for the quality assessment of the included studies

The quality assessment using NOS was assessed by two independent investigators. Most studies were considered to have a moderate (56.3%) risk of bias, and all studies were included in the assessment due to their quality assessment score (Fig. 2). More information on each study according to their quality assessment is available in Supplementary Material 1.

### Meta-analysis

**Fig. 3:**
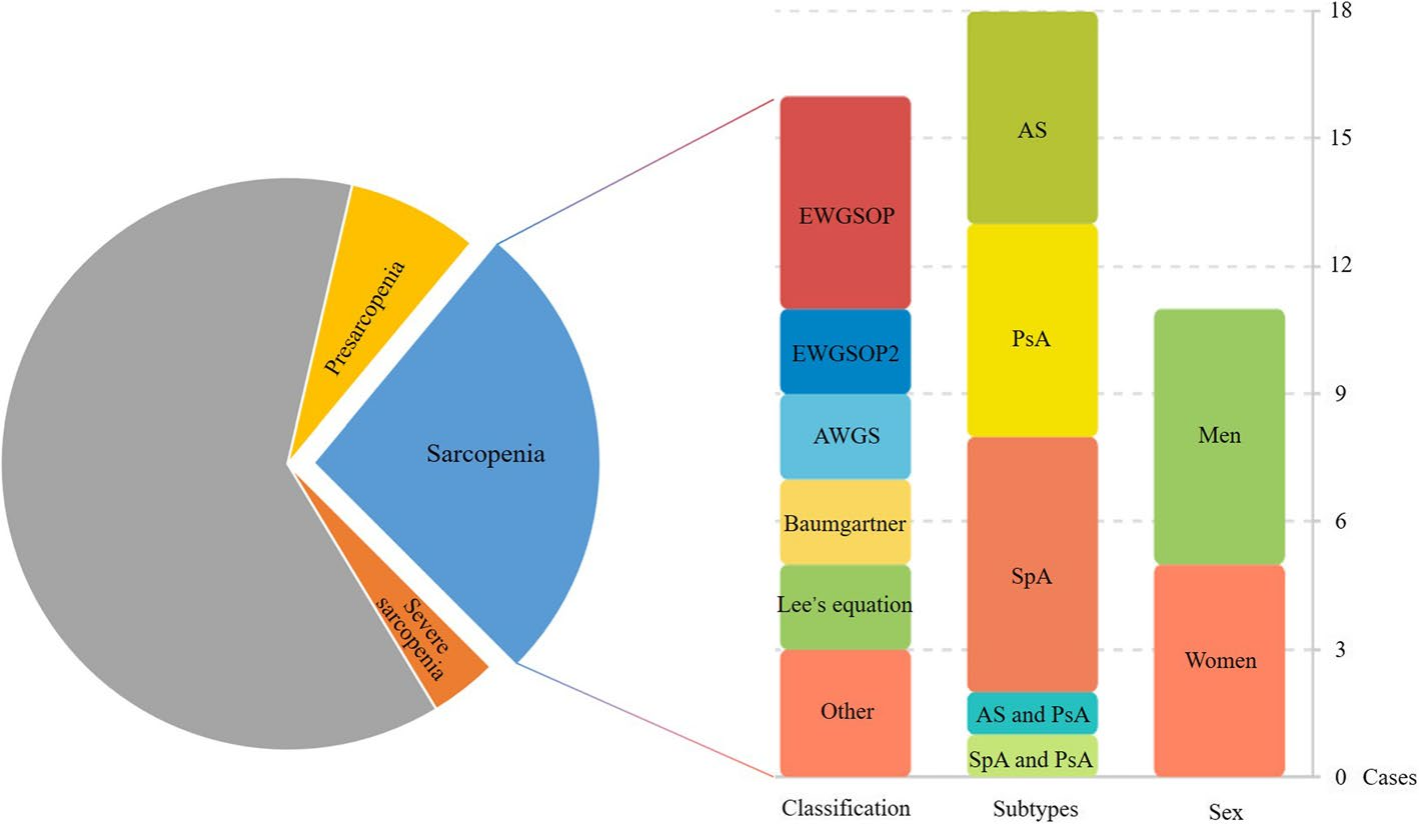
Composite pie chart for the study on sarcopenia

A total of 16 studies were included in the meta-analysis. The overall prevalence of sarcopenia, presarcopenia and severe sarcopenia was 34.8%, and the individual prevalence was 25.0%, 21.0% and 8.7%, respectively (Fig. 3). The average age of all SpA patients with clear electronic records was 46.5 years old (783 patients, SD: 13.1). The patients had a mean BMI of 26.9 (480 patients, SD: 5.3), a mean disease duration of 11.0 years (627 patients, SD: 10.0), a mean grip strength of 30.1 kg (153 patients, SD: 11.9) and a mean gait speed of 0.9 m/s (180 patients, SD: 0.3). Subgroup analyses were performed only for sarcopenia (Fig. 3). For the subgroup analyses based on classification, there were 5 studies in EWGSOP, 2 in EWGSOP2, AWGS, Baumgartner, Lee’s equation, respectively and 3 in other criteria. For SpA subtypes, there were 5 studies in AS, 5 in PsA, 1 in AS and PsA, 1 in SpA and PsA. Among the other six studies included in the analysis, the subtypes of SpA were not clearly specified. Regarding sex, we included 6 studies involving male participants and 5 studies involving female participants.

### Prevalence of sarcopenia, presarcopenia and severe sarcopenia

**Fig. 4:**
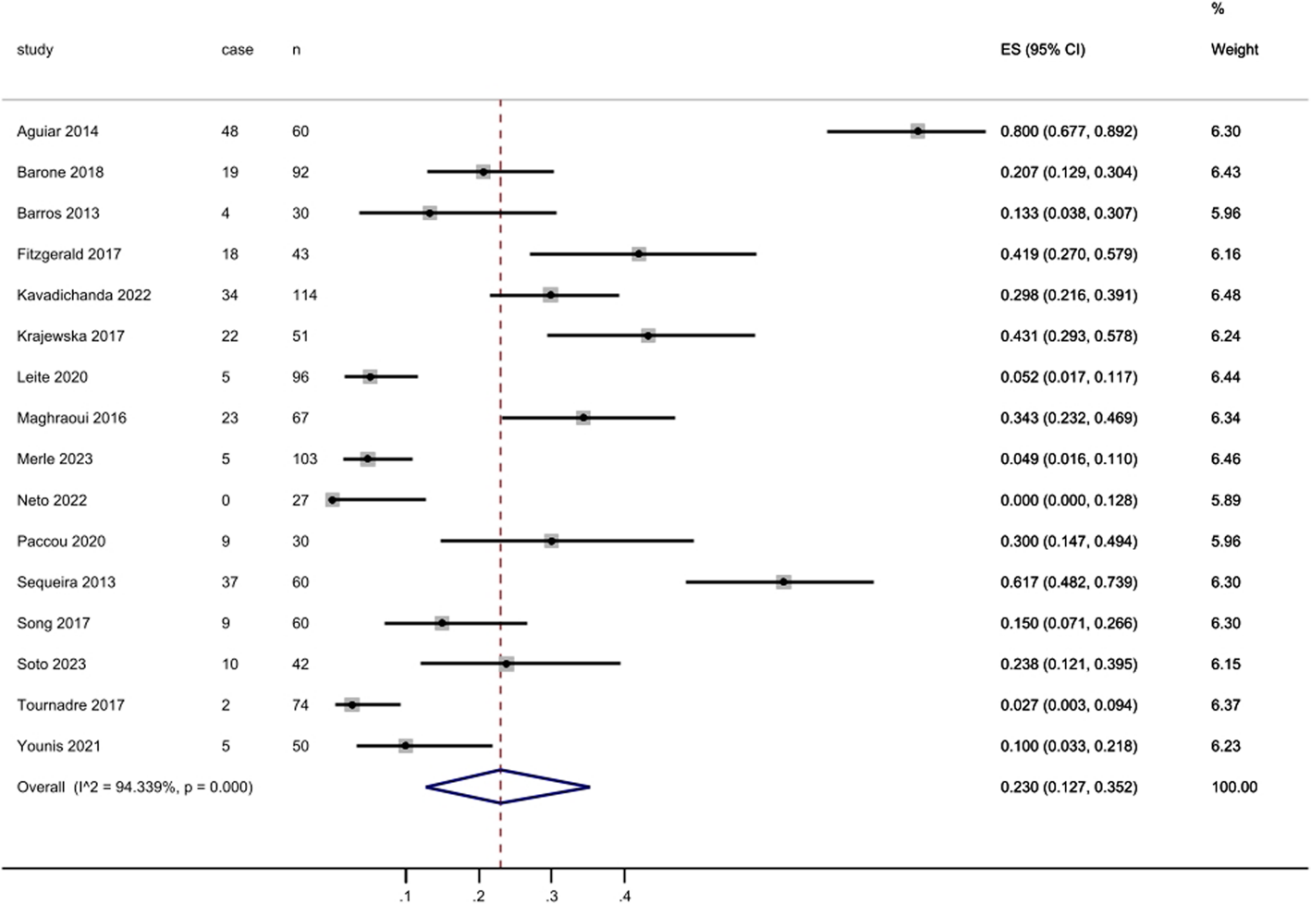
Prevalence of sarcopenia

Sarcopenia was assessed in 16 studies, and the prevalence ranged from 0 to 80.0%, demonstrating considerable variability across the studies (Fig. 4). For axSpA patients, Neto et al. conducted a cross section survey in the Portuguese population based EWGSOP2 diagnostic criteria by BIA. The sample size was limited (*N* = 27) and the average age was 37.0. Despite none of the individuals developed sarcopenia, axSpA patients exhibited lower total strength, lower limb strength and gait speed in comparison to the control group, indicating that young axSpA patients may experience potential muscle dysfunction. Agular et al. utilized the Lee’s equation to estimate the muscle mass index in a cohort of 60 patients diagnosed with SpA in Portugal and the prevalence is extremely high, with rates reaching 80.0%. Several limitations were identified, including a small sample size, potential confounding factors such as measurement bias, and the process of patient enrollment. It is worth noting that the average disease duration was more than 10 years. These contributing factors may collectively contribute to the high prevalence.

**Fig. 5:**
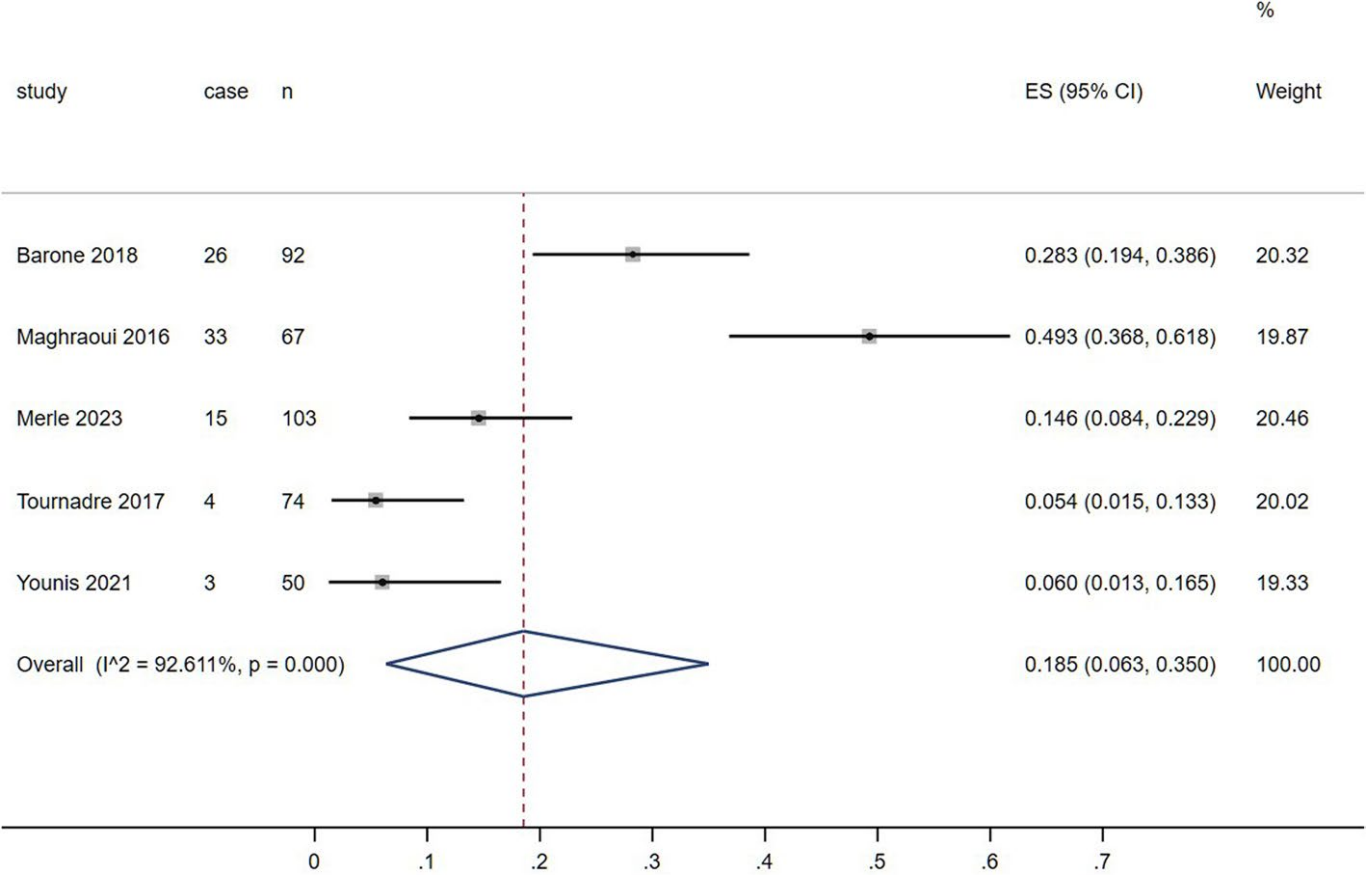
Prevalence of presarcopenia

Presarcopenia was only assessed in 5 studies, and the prevalence was 28.3%, 49.3%, 14.6%, 5.4% and 6.0%, respectively (Fig. 5). Barone et al. evaluated sarcopenia and presarcopenia in PsA, AS and rheumatoid arthritis. They found that the prevalence was approximately 20% in all the three diseases. However, there was a significant difference in the prevalence of presarcopenia between PsA and AS (*p* = 0.006). In the study conducted by Maghraoui et al., body composition and bone mineral density were evaluated using DXA in a cohort of 67 male patients with AS. The findings of the study showed that the prevalence of presarcopenia was 50.4%, sarcopenia was 34.3%, cachexia was 11.9%, and osteoporosis was 16.0% among the AS population. Merle et al. included 103 SpA patients (51% female) based on EWGSOP 2 in their study. Among the SpA patients, 15 (14.6%) had low grip strength, indicating presarcopenia. Additionally, 4.9% of SpA patients had low handgrip strength and low SMI, confirming the presence of sarcopenia. Using the established joint criteria (EWGSOP), Tournadre et al. identified sarcopenia characterized by reduced muscle mass and function in 1 out of 63 SpA patients and 1 out of 11 PsA patients, respectively. They also observed presarcopenia in 3 out of the 63 SpA patients and 1 out of the 11 PsA patients. Younis et al. enrolled 50 Iraqi AS patients with a prevalence of 10% sarcopenia and a prevalence of 6% presarcopenia.

**Fig. 6:**
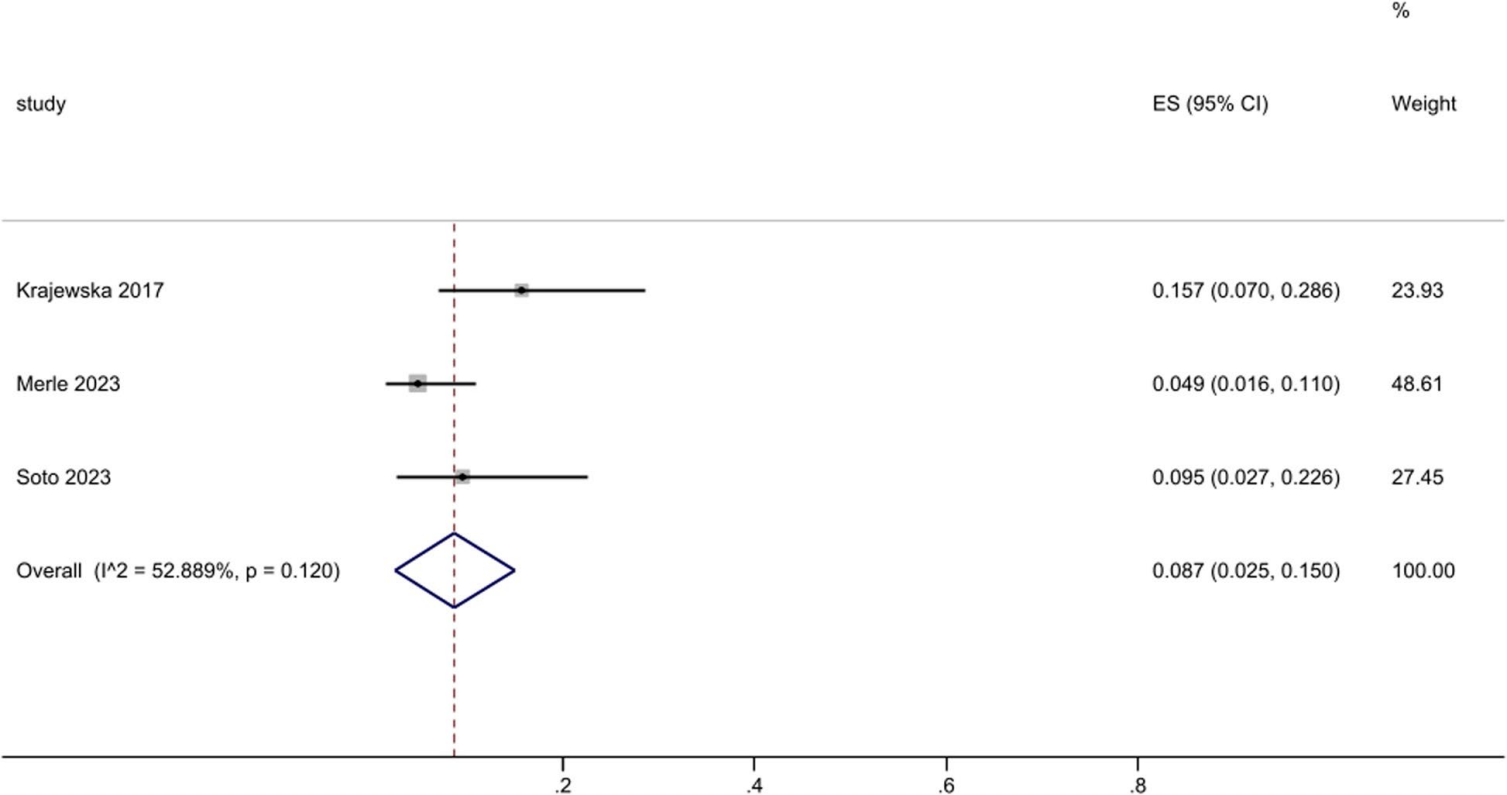
Prevalence of severe sarcopenia

Severe sarcopenia was only assessed in 3 studies, and the prevalence was 15.7%, 4.9% and 9.5%, respectively (Fig. 6). Krajewska et al. included 51 PsA women aged 50 to 75 years old. Muscle mass and lean mass were measured by BIA and diagnosed by skeletal muscle index (SMI). The prevalence of sarcopenia and presarcopenia in PsA women was 43.1% and 15.7%, respectively. Based on EWGSOP 2, a total of 103 SpA patients were included in the study by Merle et al. And the results showed that the prevalence of presarcopenia was 14.6%, while the prevalence of sarcopenia and severe sarcopenia was both 4.9%. Soto et al. enrolled 42 SpA patients and determined a prevalence of sarcopenia of approximately 33% using BIA (10/14 for sarcopenia and 4/14 for severe sarcopenia). Furthermore, the study also identified the feasibility of using ultrasound to assess sarcopenia in SpA patients.

### Subgroup analysis in sarcopenia

**Fig. 7:**
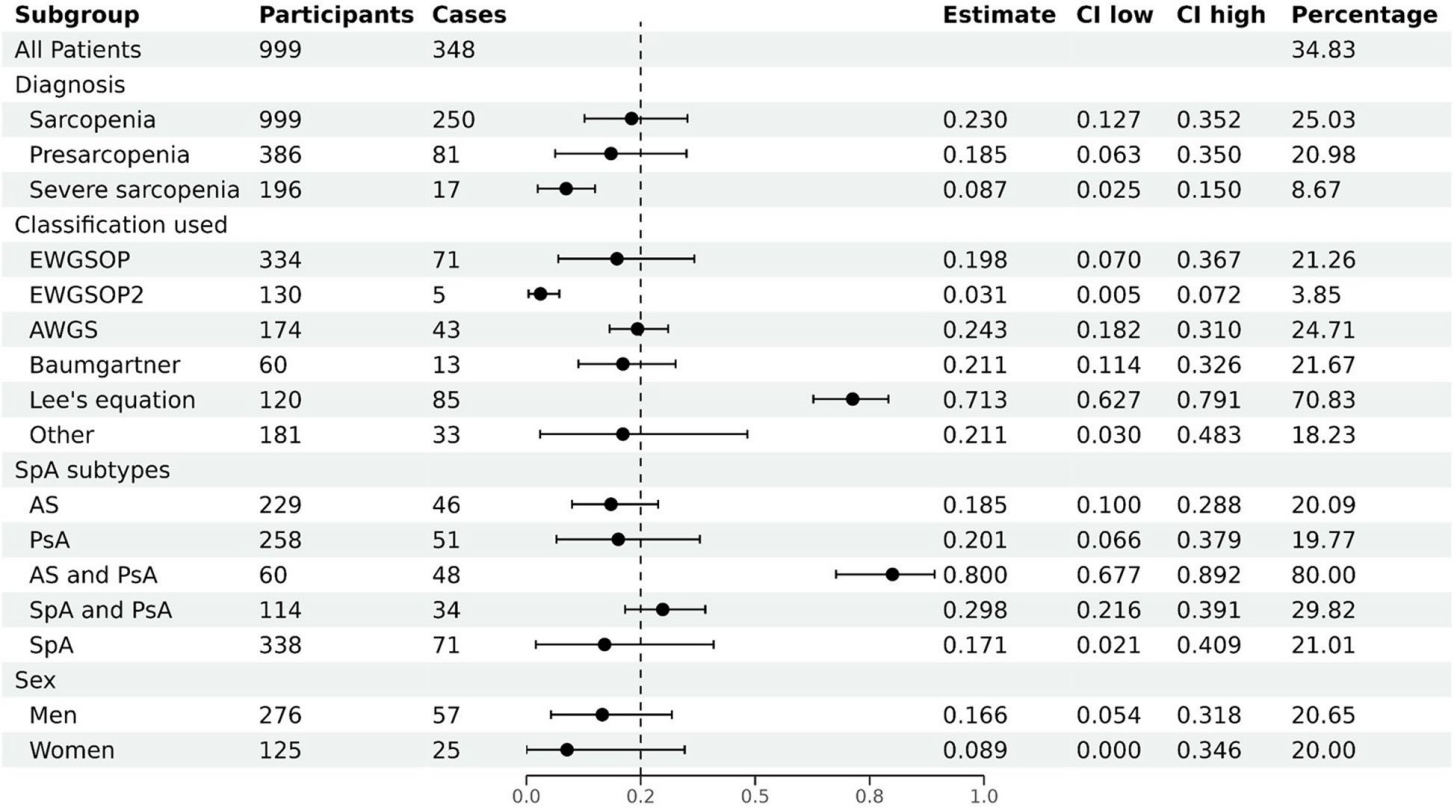
Forest plot for prevalence of sarcopenia in all studies

There were 16 studies evaluated the prevalence of sarcopenia and contained enough data had been included in subgroup analysis as mentioned previously (Fig. 7). These studies comprised of 999 SpA patients and 250 sarcopenia patients. High heterogeneity between the studies was found with an inconsistency (I2) of 94.3% and *p* < 0.000. Consequently, a random model was chosen. The prevalence for each classification was as follows: 21.3% in EWGSOP (95% CI: 0.070 to 0.367), 3.9% in EWGSOP2 (95% CI: 0.005 to 0.072), 24.7% in AWGS (95% CI: 0.182 to 0.310), 21.7% in Baumgartner (95% CI: 0.114 to 0.326), 70.8% in Lee’s Eq. (95% CI: 0.627 to 0.791) and 18.2% in others (95% CI: 0.030 to 0.483). For the subgroup analyses based on SpA subtypes, the prevalence was as follows: 20.1% in AS (95% CI: 0.100 to 0.288), 19.8% in PsA (95% CI: 0.066 to 0.379), 80.0% in AS and PsA (95% CI: 0.677 to 0.892), 29.8% in SpA and PsA (95% CI: 0.216 to 0.391) and 21.0% in SpA (95% CI: 0.021 to 0.409). Besides, the prevalence by sex was 20.7% in men (95% CI: 0.054 to 0.318) and 20.0% in women (95% CI: 0.000 to 0.346).

### The relationship between classifications and prevalence of sarcopenia

**Fig. 8:**
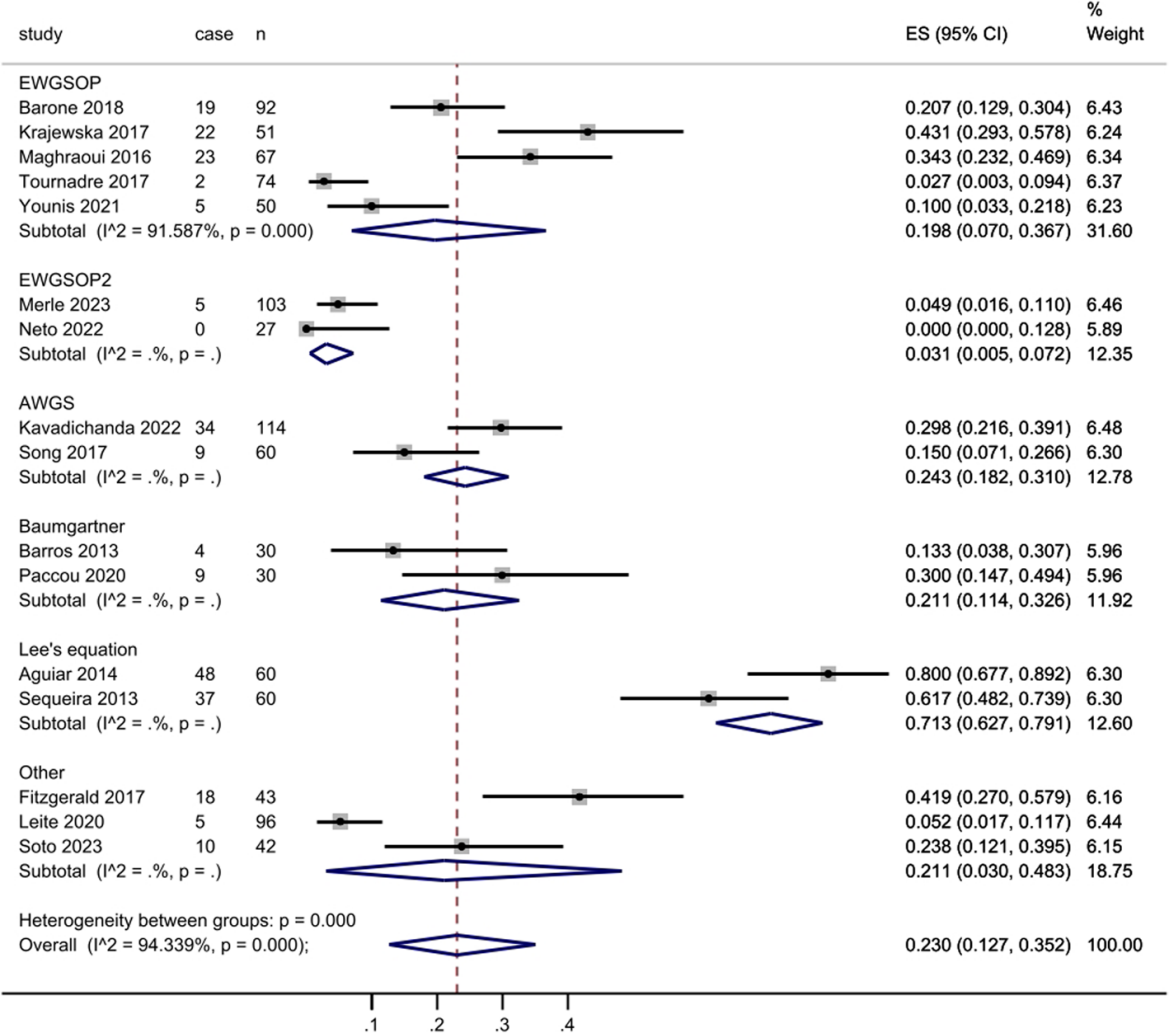
Prevalence of sarcopenia according to the classification used

The diagnostic criteria for sarcopenia vary among different working groups, but typically include three main aspects: muscle mass, muscle strength, and physical function. In these 16 included studies, multiple diagnostic criteria were utilized (Fig. 8). Results from the subgroup analysis showed a prevalence of sarcopenia of 25.0% (95% CI: 0.127 to 0.352). Group in EWGSOP had an inconsistency (I2) of 91.6% (95% CI: 0.070 to 0.367) and *p* < 0.001. The range of sarcopenia prevalence according to classifications was 2 to 31.4% in EWGSOP, 0 to 4.9% in EWGSOP2, 15.0 to 29.8% in AWGS, 13.3 to 30.0% in Baumgartner, 61.7 to 80.0% in Lee’s equation and 5.2 to 41.9% in other classifications. Overall, the prevalence determined by EWGSOP or EWGSOP2 was relatively low, whereas the prevalence determined by Lee’s equation exhibited a substantial increase. Conversely, the prevalence determined by AWGS or Baumgartner was closely approximated the overall prevalence.

### The relationship between SpA subtypes and prevalence of sarcopenia

SpA is a collection of chronic inflammatory diseases rather than a singular specific disease. The subtypes analysis of SpA included AS and PsA in 9 studies, one of them had AS and PsA in the same article conducted by Barone et al. Furthermore, Aguiar et al. conducted their study by combining AS and PsA as a whole group. Similarly, Kavadichanda et al. grouped together SpA and PsA in their study. In the remaining 6 studies, the subtypes of SpA were not specifically classified. Therefore, they were collectively classified as SpA.

**Fig. 9:**
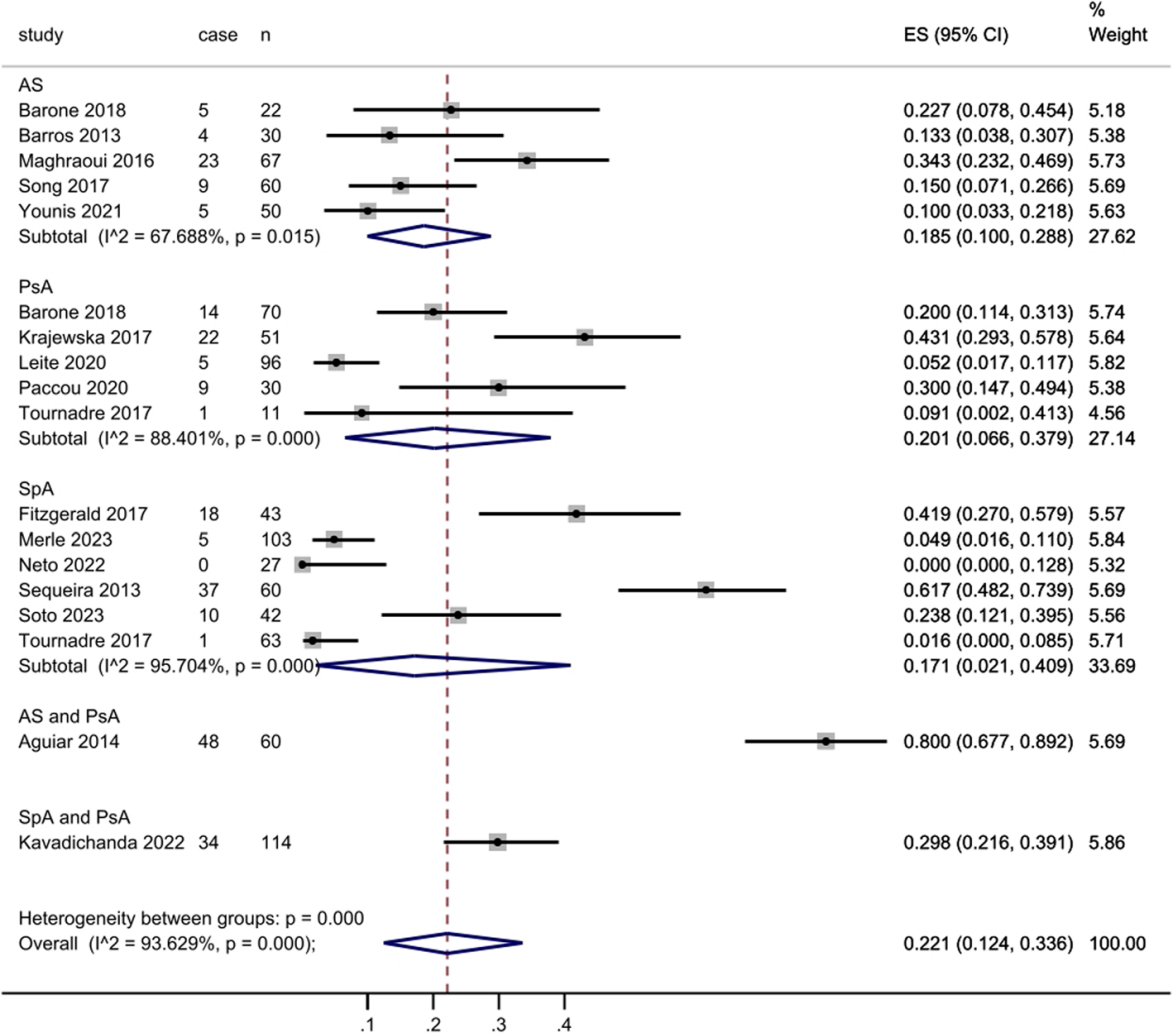
Prevalence sarcopenia according to the subtype of SpA.

To be specific, the sample for these studies consisted of 999 patients diagnosed with SpA and 250 patients diagnosed with sarcopenia, and the overall prevalence was still 25.0% (Fig. 9). High heterogeneity between the studies was found with an inconsistency (I2) of 93.6% (95% CI: 0.124 to 0.336) and *p* < 0.000. The range of sarcopenia prevalence according to subtypes was 10.0–34.3% in AS (inconsistency (I2): 67.7%, 95% CI: 0.100 to 0.288, *p* = 0.015), 5.2–43.1% in PsA (inconsistency (I2): 88.4%, 95% CI: 0.066 to 0.379, *p* < 0.000), 80.0% in AS and PsA (95% CI: 0.677 to 0.890), 29.8% in SpA and PsA (95% CI: 0.216 to 0.391), 0 to 61.7% in SpA (inconsistency (I2): 95.7%, 95% CI: 0.021 to 0.409, *p* < 0.000).

### The relationship between sex and prevalence of sarcopenia

**Fig. 10:**
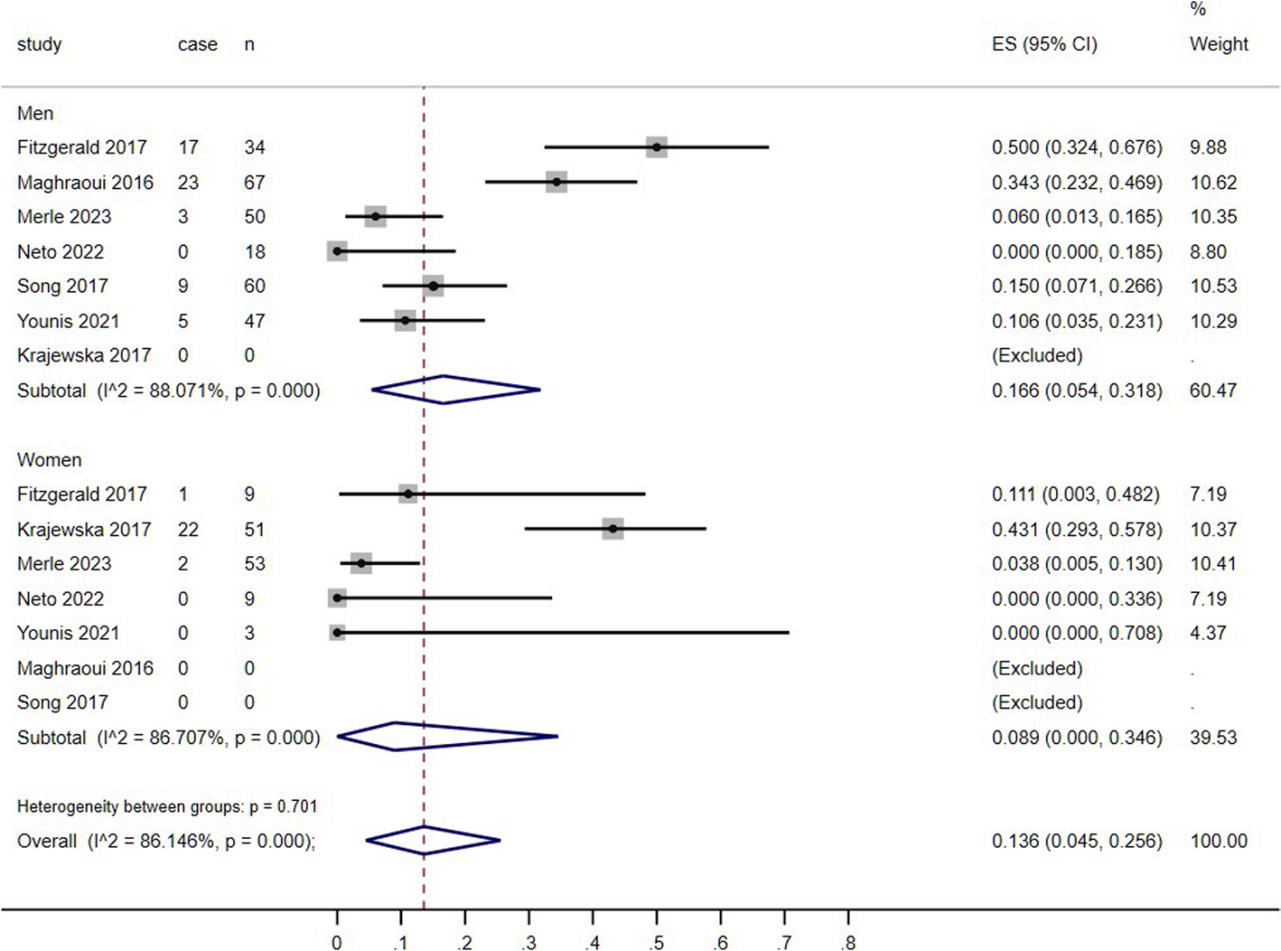
Prevalence of sarcopenia according to the sex

SpA typically affects males during their young and middle-aged years. Therefore, conducting subgroup analyses based on sex is of utmost importance. When the data was stratified by sex, it was found that only 6 studies included sufficient data for men, while 5 studies included data for women (Fig. 10). These studies consisted of 401 patients diagnosed with SpA and 82 patients diagnosed with sarcopenia. High heterogeneity between the studies was found with an inconsistency (I2) of 86.1% (95% CI: 0.045 to 0.256) and *p* < 0.000. The overall prevalence of sarcopenia was 20% in men and 20.7% in women. Group in men had an inconsistency (I2) of 88.1% (95% CI: 0.054 to 0.318) and *p* < 0.000 with the range of sarcopenia prevalence from 0 to 50.0%, and group in women had an inconsistency (I2) of 86.7% (95% CI: 0 to 0.346) and *p* < 0.000 with the range of sarcopenia prevalence from 0 to 43.1%. Overall, there was no significant difference in the prevalence between men and women.

## Discussion

SpA is a chronic inflammatory disease that includes several subtypes, and sarcopenia is a progressive and complex disease that may be associated with it. Sarcopenia has been found to be strongly associated with a range of adverse outcomes, including increased disability, reduced quality of life, and higher mortality rates, even amone young individuals [30]. Therefore, exploring the prevalence of sarcopenia in SpA patients has important clinical implications, and then identifying and managing it is crucial for improving overall health outcomes. The purpose of this study is to gather and analyze all existing research on the prevalence of sarcopenia in SpA patients in order to provide a more accurate estimate of the condition’s occurrence within this specific disease. To our knowledge, this is the first systematic review and meta-analysis that investigates the overall prevalence of sarcopenia, presarcopenia and severe sarcopenia in SpA patients. Due to the significant clinical, methodological, and statistical heterogeneity observed among the included studies, we did not conduct the publication bias and sensitivity analysis. It is worth mentioning that certain studies reported a prevalence of zero for sarcopenia [22, 27], therefore we utilized the ‘metaprop’ to address this issue. Furthermore, it is important to note that some subgroups had a limited number of studies (1 or 2), which precluded the heterogeneity test.

Our study included 16 studies and 999 SpA patients that met the inclusion criteria. The overall prevalence ranged from 0 to 80.0% in sarcopenia, 5.4 to 49.3% in presarcopenia and 4.9 to 15.7% in severe sarcopenia. The findings suggest that sarcopenia is more prevalent in SpA compared to control groups in most of the studies. Compared to the prevalence of general sarcopenia without any other diseases (10–27%) [31], risk of muscular atrophy in SpA is increased to some extent. Furthermore, our meta-analysis revealed a notable trend and suggested that individuals with sarcopenia in SpA tend to be of advanced age (42.3 vs. 40.9 [21], 32.6 vs. 36.6 [28]), have a longer duration of the disease (11.6 vs. 9.3 [21], 12.4 vs. 6.9 [28]) and a lower BMI (21.6 vs. 25.3 [21], 24 vs. 28.8 [17], 20.1 vs. 28.4 [28]). Even in the case of presarcopenia, we can still observe this trend in the demographics [21]. Especially, in the study conducted by Neto et al., none of the participants were found to have sarcopenia. However, these patients still experienced reduced physical performance and lower strength than the healthy controls, despite having normal muscle mass. This suggesting the presence of possible muscle dysfunction [22]. Besides, in the cohort study, SpA patients underwent medical intervention, resulting in a decrease in disease activity. Additionally, an increase in muscle mass was observed [16, 23]. However, it remains unclear whether the improvement should be attributed to the direct effect of the medication or the indirect effect following improvement in symptoms. According to a study on global prevalence of sarcopenia, the average age was 68.5 years old [31]. In contrast, our study found that the average age of sarcopenia in SpA patients was significantly younger. All of these findings hightlight the importance for clinicians to carefully consider these risk factors when assessing and managing sarcopenia in SpA.

As we known, sarcopenia is influenced by various factors, such as age, sex, level of inflammation, disease duration, BMI, and treatment [3]. In this study, we collected patient characteristics including age, sex, disease duration, BMI, and sample size. In addition to assessing muscle mass, muscle strength, and physical performance, we also gathered information on sarcopeniat classification, measurement methods, and correction methods, as presented in Table 2. However, it is unfortunate that this part of the content is quite incomplete. Consequently, we were unable to further adjust for risk factors such as age and sex. And we strongly encourage follow-up studies to provide detailed explanations of these relevant factors.

The prevalence of sarcopenia varied between regions. Most of studies were conducted in Europe, with the highest prevalence rate of sarcopenia observed in Portugal/Europe (48/60) [14], while the lowest rate was also reported in Portugal/Europe (0/27) [22]. This may be related to limited studies conducted in other regions, like Asia and Africa. In measuring muscle mass, DXA was found to be the most commonly used method, followed by BIA. Although these methods are golden rules for sarcopenia, considering the presence of limited medical conditions in reality, more and more researchers have begun exploring the role of computed tomography, magnetic resonance imaging and ultrasonography in measuring muscle mass. In our systematic review, the study conducted by Soto et al. identified the potential of ultrasound in diagnosing sarcopenia in SpA [26]. The AWGS had even suggested the concept of “potential sarcopenia” which can be identified through simple methods such as finger-ring test [11]. Subsequently, this can guide healthcare practitioners to conduct more specialized examinations, making it applicable for primary healthcare facilities and aiding in early intervention. In conclusion, diagnostic tools may have a significant impact on the detection rate of sarcopenia.

Our study included 5 major classifications of sarcopenia with different cut-off points and instruments to assess muscle mass, the most commonly reported classification of them was the EWGSOP, followed by the EWGSOP2, AWGS, Baumgartner and Lee’s equation. Meanwhile, we estimated that the overall prevalence of sarcopenia was 21.3% in EWGSOP, 3.8% in EWGSOP2, 24.7% in AWGS, 21.7% in Baumgartner and 70.8% in Lee’s equation. The first three diagnostic criteria are widely recognized. However, the diagnostic criteria proposed by Baumgartner in 1998, which defines sarcopenia as a relative SMI less than 2 standard deviations from healthy individuals of the same sex, has its limitations. This method, which evaluates only muscle mass, lacks a comparative measure of muscle strength that is included in other contemporary diagnostic criteria for sarcopenia. Despite this, due to its simplicity and ease of use, it remains a popular choice for epidemiological investigations into sarcopenia. Lee et al. developed a predictive model that primarily incorporates measurements of limb circumference and skinfold thickness, with adjustments made for variables such as sex, age, and ethnicity [13]. This model has been validated in both non-obese and obese populations. However, its application remains limited due to the scarcity of related studies. In contrast, the diagnostic criteria proposed by the EWGSOP and AWGS are more comprehensive. They consider three key factors: loss of muscle mass, decreased muscle strength and reduced physical performance, each with their respective cut-off values, as shown in Table 1. This multifaceted approach provides a more holistic evaluation of sarcopenia. These variations in diagnostic criteria may also contribute to the disparities in the prevalence of sarcopenia.

In this study, the lack of primary data from various studies prevented the establishment of a uniform classification, which may account for the differences in prevalence observed between different studies. For instance, Song et al. conducted a study in South Korea involving 60 patients with AS, utilizing both the AWGS and Korea criteria. They found that the prevalence of sarcopenia was 15% and 16.7%, respectively, resulting in a subtle difference [25]. Furthermore, as mentioned earlier, the previous classification such as Baumgartner and Lee’s equation have their limitations, and there may be a certain disparity between the classification widely accepted in recent years. Unfortunately, standardization these studies in assessing the prevalence of sarcopenia in SpA is currently not possible. However, this emphasizes the importance of recognizing the impact of ongoing improvements in classification on sarcopenia prevalence in real-world settings.

It is well known that SpA has various subtypes, but there has been no research comparing the prevalence of sarcopenia among different subtypes. After conducting an initial search, we found three studies related to sarcopenia in JIA [32–34]. Unfortunately, these articles did not provide specific and clear information regarding the subtypes of JIA included. Only one article mentioned the inclusion of various subtypes of JIA. As a result, we decided not to include these articles in our study. However, the JIA group showed a surprisingly high prevalence of sarcopenia, reaching 71.4%. In contrast, the prevalence of sarcopenia in the subtypes of AS or PSA was relatively lower and have no significant differences (20.1% vs. 19.8%), which may be attributed to the larger number of patients included. Furthermore, several studies had shown a correlation between the risk of sarcopenia and disease activity. However, additional research is required to validate these findings using a larger sample size and to explore the factors that influence the development of sarcopenia in SpA patients. Future research that investigates the differences in sarcopenia status among these subtypes could provide valuable insights into the shared pathophysiology between sarcopenia and SpA.

In previous studies, men are more commonly affected in SpA patients, while women are more commonly affected by sarcopenia [35]. However, many studies did not provide specific information regarding sex classification. In our study, 6 articles specifically described the prevalence of sarcopenia in men, while five articles specifically focused on the prevalence of sarcopenia in women. In fact, some studies only included male participants, while others only included female participants. Overall, sex differences in the prevalence of sarcopenia among SpA patients had not been observed (men: 20.7% vs. women: 20%) and several factors may contribute to it, like hormonal differences, physical activity, nutritional status and biological factors, all of them could influence the development of sarcopenia [35, 36]. Further research is needed to better understand the role that sex plays in the development of sarcopenia and to establish appropriate interventions to improve it in both men and women patients in SpA.

By conducting a systematic review and meta-analysis, we obtained a more robust and reliable understanding of the prevalence of sarcopenia in SpA by pooling data from multiple studies. Our findings demonstrate that sarcopenia is prevalent in SpA, generally higher than estimates previously reported from the general population. The detrimental effects of sarcopenia, such as an increased risk of falling and fractures, impairment of independence and quality of life, and the likelihood of mobility disorders, have been well documented. This highlights the importance of early clinical assessment and interventions to prevent adverse outcomes associated with muscle atrophy in SpA patients. Addressing the components of presarcopenia at an early stage could have benefits in preventing and reversing sarcopenia. In terms of treatment, resistance training and nutritious supplementary have been widely agreed in managing sarcopenia [37, 38], while further research is needed to establish their specific benefits in SpA.

### Limits

It is important to acknowledge the limitations of our study. Firstly, the studies included in our analysis had varying definitions and cut-offs for sarcopenia, leading to heterogeneity in our findings. Additionally, the criteria used for diagnosing sarcopenia may not be appropriate for the specific disease, future research should aim to establish standardized definitions and criteria for diagnosing sarcopenia in SpA. Secondly, the majority of the studies included in our analysis were cross section surveys, which limited our ability to investigate the cause and effect relationship between SpA and sarcopenia. Longitudinal studies are needed to better understand the relationship between sarcopenia and the progression of SpA over time. Thirdly, it is important to consider that different types of SpA may exhibit distinct characteristics. Factors such as age, sex, level of inflammation, disease duration, BMI, and treatment may influence the prevalence and severity of sarcopenia. Future studies should take these factors into account to investigate their impact on sarcopenia in SpA.

## Conclusion

In conclusion, our study highlights the relatively high prevalence of sarcopenia in SpA. This emphasizes the importance of implementing routine screening and targeted interventions to effectively manage sarcopenia in SpA patients, as it can have a significant impact on their overall health and quality of life. Additionally, our findings underscore the need for further research in specific areas, such as investigating the relationship of sarcopenia on disease progression and outcomes. Future studies should focus on longitudinal designs, identifying potential risk factors, and exploring the underlying mechanisms and potential interventions for sarcopenia in SpA patients.

### Supplementary Information

**Supplementary Material 1.**

## Data Availability

No datasets were generated or analysed during the current study.

## Abbreviations

AS: Ankylosing spondylitis
ASQoL: Ankylosing Spondylitis quality of life questionnaire
AWGS: Asian Working Group for Sarcopenia
BIA: Bioelectric impedance analysis
BMI: Body mass index
CI: Confidence interval
DXA: Dual energy X-ray absorptiometry
EWGSOP: European Working Group on Sarcopenia in Older People
GPPAQ: General practice physical activity questionnaire
HAQ: Health assessment questionnaire
IPAQ: International physical activity questionnaire
JIA: Juvenile idiopathic arthritis
NOS: Newcastle-Ottawa Scale
PRISMA: Preferred Reporting Items for Systematic Review and Meta-Analysis
PROSPERO: International Prospective Register of Systematic Reviews
PsA: Psoriatic arthritis
SarQoL: Sarcopenia and quality of life
SMI: Skeletal muscle index
SpA: Spondyloarthritis
TNF: Tumor necrosis factor
